# Machine learning-based coronary heart disease diagnosis model for type 2 diabetes patients

**DOI:** 10.3389/fendo.2025.1550793

**Published:** 2025-05-22

**Authors:** Yingxi Chen, Chunyu Wang, Xiaozhu Liu, Minjie Duan, Tianyu Xiang, Haodong Huang

**Affiliations:** ^1^ Department of Anatomy, Institute of Neuroscience, Chongqing Medical University Basic Medical College, Chongqing, China; ^2^ Department of Pediatrics, West China Second University Hospital, Sichuan University, Chengdu, China; ^3^ Department of Critical Care Medicine, Beijing Shijitan Hospital, Capital Medical University, Beijing, China; ^4^ Chevidence Lab of Child and Adolescent Health, Children’s Hospital of Chongqing Medical University, Chongqing, China; ^5^ Information Center, The University-Town Hospital of Chongqing Medical University, Chongqing, China; ^6^ Department of Obstetrics and Gynecology, Women and Children’s Hospital of Chongqing Medical University, Chongqing, China; ^7^ Chongqing Municipal Health Commission Key Laboratory of Perinatal Medicine, Chongqing, China

**Keywords:** machine learning, type 2 diabetes mellitus, coronary heart diseases, diagnosis model, diabetic comorbidities

## Abstract

**Background:**

To establish a classification model for assisting the diagnosis of type 2 diabetes mellitus (T2DM) complicated with coronary heart disease (CHD).

**Methods:**

Patients with T2DM who underwent coronary angiography (CA) were enrolled from seven affiliated hospitals of Chongqing Medical University. Statistical differences in clinical variables between T2DM with or without CHD patients were verified using univariate analysis. The original data was divided into a training set and a validation set in a 7:3 ratio. The training set data were used to screen features using Logistic regression, Lasso regression, or recursive feature elimination (RFE). Five machine learning algorithms, including Logistic regression, Support Vector Machine (SVM), Random Forest (RF), eXtreme gradient boosting (XgBoost), and Light Gradient Boosting Machine (LightGBM), were selected for modeling. The performance of the models was verified through 5-fold cross-validation and the training set.

**Results:**

Clinical data were collected from 1943 patients with T2DM complicated with CHD and 574 T2DM patients without CHD. Univariate analysis identified 20 optimal risk factors, four of the risk factors had over 30% missing values, we ultimately included 16 risk factors. Logistic regression screened eight features, Lasso regression screened ten features, the RFE method screened eight, fourteen, sixteen, and thirteen features for SVM, RF, XgBoost, and LightGBM, respectively. Among all models, the XgBoost model based on features selected by RFE+LightGBM demonstrated the best performance, achieving an AUC of 0.814 (95% CI, 0.779-0.847), accuracy of 0.799 (95% CI, 0.771-0.827), precision of 0.841 (95% CI, 0.812-0.868), recall of 0.920 (95% CI, 0.898-0.941), and F1-score of 0.879 (95% CI, 0.859-0.897) in the testing set.

**Conclusions:**

Based on T2DM data and machine learning theory, a Bayesian-optimized XgBoost model was established using the RFE+LightGBM method. This model effectively determines whether T2DM patients have CHD.

## Graphical Abstract

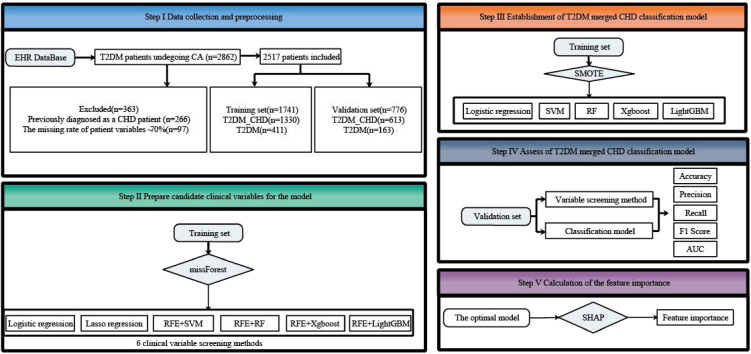

## Introduction

Type 2 diabetes mellitus (T2DM) is a chronic metabolic disease characterized by insufficient insulin production, poor insulin action, or both. According to the 11th edition of the International Diabetes Federation (IDF) Diabetes Atlas published in 2025, approximately 589 million adults (aged 20–79 years) are living with diabetes globally, equating to nearly 1 in 9 adults. Alarmingly, diabetes was responsible for 3.4 million deaths in 2024, translating to one death every six seconds (1). A nationwide cross-sectional study conducted in China in 2024 involving 1.87 million patients with type 2 diabetes mellitus (T2DM) found that approximately 67.5% of the participants were classified as being at very high risk for cardiovascular disease (CVD), with coronary heart disease (CHD) identified as the most common cardiovascular complication (2). Furthermore, data from the CAPTURE study indicated that 33.9% of Chinese adults with T2DM had established CVD, with CHD being the most prevalent subtype, affecting 16.0% of the study population (3).

T2DM significantly increases the risk of developing coronary heart disease (CHD), largely due to a complex interplay of metabolic and vascular dysfunction. Persistent hyperglycemia promotes the formation of advanced glycation end-products (AGEs), which impair endothelial function and promote inflammation (4). Additionally, insulin resistance and associated dyslipidemia accelerate atherosclerosis through increased oxidative stress and macrophage activation, contributing to plaque formation and vascular injury (5). Chronic systemic inflammation and altered adipokine profiles further exacerbate vascular dysfunction in T2DM patients (6).

Although there is ongoing debate about whether diabetes mellitus (DM) precedes coronary CHD or coexists early in the disease, it is widely accepted that DM-induced oxidative stress, advanced glycosylated end-products, and chronic inflammatory responses harm vascular endothelial function and cause cardiovascular disease (7, 8). This suggests that T2DM is a major risk factor for microvascular and macrovascular complications (9).

T2DM patients with CHD are more susceptible to severe atherosclerosis than non-DM patients with CHD, with narrower arterial lumens (10). These patients often exhibit two or three vessel lesions, presenting as multivessel and diffuse lesions (11). Symptoms of CHD in T2DM patients are often atypical compared to non-DM patients due to severe autonomic disorders in T2DM patients, which increase pain thresholds. Consequently, T2DM patients may only experience mild pain or no pain, even during severe myocardial ischemia (12).

Coronary angiography (CA) is a necessary and widely used method for diagnosing CHD in clinical practice (13). However, CA is invasive and can cause complications such as arterial dissection, arrhythmia, and even death. Additionally, image-based detection techniques are expensive and not suitable for screening large populations or for patient follow-up. Given the drawbacks of CA, several non-invasive examination methods are widely used in clinical settings, including coronary computed tomography angiography, cardiac Magnetic Resonance Imaging (MRI), Holter monitoring, and echocardiography. As a non-invasive method, computed tomography angiography offers high sensitivity and negative predictive value, but there is still debate over whether accurate judgments can be made based solely on imaging examination results (14). Cardiac MRI can noninvasively examine cardiovascular morphology, ventricular function, myocardial perfusion, tissue characteristics, blood flow quantification, and coronary artery disease. However, patients with metal implants (such as defibrillators, pacemakers etc.) cannot undergo MRI, limiting its clinical implementation to only 13.5% (15). Holter monitoring is a commonly used non-invasive method for detecting cardiovascular diseases. It offers advantages such as real-time monitoring, repeatability, and affordability. However, the Electrocardiogram (ECG) signals obtained from Holter monitors are susceptible to external environmental influences (16). In addition, many T2DM patients with CHD do not show abnormal electrocardiograms in the early stages. Once the Holter monitor detects abnormal signals, it indicates that CHD has already reached a relatively serious stage. Therefore, there is an urgent need to find new auxiliary methods for the early diagnosis of CHD in T2DM patients.

In this study, we retrospectively collected electronic medical record data from T2DM patients who underwent CA using the medical data platform of Chongqing Medical University. Based on this data and relevant machine learning theory, we developed a Bayesian-optimized eXtreme gradient boosting(XgBoost) model using the recursive feature elimination+Light Gradient Boosting Machine (RFE+LightGBM) method. This model effectively diagnoses whether T2DM patients also have CHD.

## Methods

### Study population

We retrospectively enrolled electronic medical record data of T2DM patients who underwent CA from the medical data platform of Chongqing Medical University affiliated hospitals, China. All data had been desensitized. The study included patients discharged between January 1st, 2015, and December 31st, 2021.

The inclusion and exclusion criteria were as follows. Inclusion criteria: Patients who met the diagnostic criteria for T2DM according to Chinese Guidelines for the Prevention and Treatment of T2DM (2020) (17), or those with a documented history of T2DM duration; patients who underwent CA during hospitalization and had complete surgical records. Exclusion criteria: Patients with a history of CHD, type 1 DM, gestational DM, acute complications of DM, autoimmune diseases such as systemic lupus erythematosus and rheumatic heart disease, severe organ failure, or malignant tumors; patients with a clinical data deletion rate exceeding 70%.

### Data collection

A total of 2862 T2DM patients were initially enrolled based on the inclusion criteria. Exclusions included 266 patients previously diagnosed with CHD and 79 patients with a clinical data deletion rate exceeding 70%. Ultimately, 2517 T2DM patients were included. Among them, 1943 patients were classified into the T2DM with CHD group (T2DM_CHD) based on significant stenosis (≥50%) in one or more branches of the left main trunk, anterior descending branch, circumflex branch, or right coronary artery, as documented in their surgical records. The remaining 574 patients formed the T2DM group. These two groups were divided into a training set (T2DM_CHD group:1330, T2DM group:411) and a testing set(T2DM_CHD group:613, T2DM group:163) in a 7:3 ratio (as shown in Step I of the Graphical Abstract). The training set was used for feature selection and model development, while the testing set was used to evaluate the performance of the established classification models.

### Features selection and data preprocessing

We collected a total of 48 clinical variables (**Supplementary Table 1**), referred to as “risk factors” in CHD, based on features specific to T2DM combined with CHD and CHD clinical guidelines (18). After screening, clinical risk factors with a missing percentage of less than 30% were retained and imputed using the Random Forest (RF) method (implemented in R using the *missForest* packages). Statistical differences in these risk factors between the groups were verified using univariate analysis. Values were assigned to non-digitized risk factors as shown in **Supplementary Table 2**. Measurement data were analyzed using t-tests or Mann-Whitney U tests, while enumeration data were analyzed using the Chi-square test (χ^2^ test). A significance level of *P*<0.05 was considered. Six clinical variable screening methods were employed, including Logistic regression (using the *stats* packages in R), Lasso regression (using the *glmnet* packages in R), Support Vector Machine (SVM), RF, XgBoost, and LightGBM based on RFE using python packages (*sklearn*). These methods used to select significant features and prepare for establishing classification models.

### Model establishment and performance assessment

In clinical research, positive and negative samples are often imbalanced, and classification models established using imbalanced samples cannot effectively predict diseases. The imbalances in the training set were addressed using the Synthetic Minority Over-Sampling Technique (SMOTE, implemented with the *imblearn* package in Python). The classification model algorithms were optimized using the Bayesian optimization algorithm (implemented with the *bayesian-optimization* package in Python). Classification models for diagnosing CHD in T2DM patients were established using various underlying models, including Logistic Regression, SVM, RF, XgBoost, and LightGBM. The classification modeling was performed using Python’s *sklearn* package.

The testing set was used to assess the performance of the classification model. Evaluation metrics included accuracy, precision, recall, F1 score, and AUC (with 95% confidence intervals calculated using the Bootstrap resampling method).

### Calculation of the feature importance

Machine learning models are often criticized for their lack of interpretability, particularly in explaining why an algorithm provides an auxiliary diagnosis for a particular patient cohort. SHapley Additive exPlanations (SHAP), implemented using the *shap* package in Python, is a conventional approach that can interpret machine learning models, providing both global and local interpretability simultaneously (19). We used SHAP to visualize and calculate the importance of features from the final classification model.

## Results

### Patient characteristics

We enrolled 2517 T2DM patients who underwent CA using the medical data platform of Chongqing Medical University from January 1st, 2015, to December 31st, 2021. This cohort included 1943 T2DM_CHD patients and 574 T2DM patients. We identified 20 risk factors with statistically significant differences between T2DM patients with and without CHD were identified using univariate analysis. However, 4 of these risk factors had more than 30% missing data and were excluded (Distributions and statistical differences before and after imputation are presented in **Supplementary Figure S1** and detailed in **Supplementary Table 3**). Ultimately, we included 16 risk factors in the study (**Table 1**).

**Table 1:** Univariate analysis of related indexes in T2DM group and T2DM_CAD group.

Variables	Measurement units	T2DM_CAD (n=1943)	T2DM (n=574)	χ^2^ */Z/t*	*P*
Demographic information					
Age	year	65.50 (58.00-70.00)	55.50 (53.00-66.00)	-7.152	<0.001
Male	n (%)	1095/1943 (56.36%)	322/574 (56.10%)	0.012	0.913
BMI*	kg/m^2^	24.39 (22.77-26.56)	25.16 (24.25-26.33)	-3.245	0.001
Smoke	n (%)	739/1841 (40.14%)	143/552 (25.91%)	36.978	<0.001
Drink	n (%)	512/1832 (27.95%)	128/551 (23.23%)	4.789	0.028
Systolic pressure	mmHg	144.00 (137.00-166.00)	142.00 (130.50-168.00)	-1.707	0.088
Diastolic pressure	mmHg	87.50 (79.00-94.00)	89.50 (83.00-101.50)	-0.501	0.617
Heart rate	beats/min	82.50 (76.00-94.00)	85.00 (76.50-93.00)	-0.681	0.496
Clinical history					
Hypertension	n (%)	1423/1943 (73.24%)	370/574 (64.46%)	16.660	<0.001
Family history of diabetes	n (%)	191/1943 (9.83%)	55/574 (9.58%)	0.031	0.860
Family history of CHD	n (%)	78/1943 (4.01%)	28/574 (4.88%)	0.819	0.365
Diabetic nephropathy	n (%)	140/1943 (7.21%)	24/574 (4.18%)	6.653	0.010
Cerebral infarction	n (%)	258/1943 (13.28%)	93/574 (16.20%)	3.156	0.076
Carotid atherosclerosis	n (%)	333/1943 (17.14%)	95/574 (16.55%)	0.109	0.742
Atrial fibrillation	n (%)	78/1943 (4.01%)	34/574 (5.92%)	3.798	0.051
Heart block	n (%)	61/1943 (3.14%)	14/574 (2.44%)	0.752	0.386
Hyperlipidemia	n (%)	534/1943 (27.48%)	162/574 (28.57%)	0.121	0.728
Lab values					
Urine Glu	n (%)	704/1668 (42.21%)	140/503 (27.83%)	33.598	<0.001
Urine protein*	n (%)	316/1357 (23.29%)	65/392 (16.58%)	8.025	0.005
Urine WBC*	/µL	5.05 (2.00-20.80)	4.70 (0.35-124.85)	-2.628	0.009
Urine RBC*	/µL	5.15 (1.60-11.60)	5.75 (0.55-14.45)	-1.885	0.059
Urine Crea*	mmol/L	8.90 (4.40-17.60)	8.80 (3.40-16.50)	-0.913	0.361
ALT	U/L	23.00 (16.00-35.00)	22.00 (14.00-32.00)	-0.066	0.948
AST	U/L	22.50 (17.00-33.00)	22.00 (17.00-27.00)	-2.893	0.004
GGT	U/L	28.00 (18.00-47.00)	28.00 (19.00-44.00)	-0.738	0.461
TBIL	µmol/L	10.70 (8.10-14.30)	11.40 (8.80-14.90)	-0.169	0.866
TP	g/L	69.37 ± 7.13	70.68 ± 6.39	-3.507	<0.001
Apo Ai	g/L	1.40 (1.23-1.63)	1.50 (1.34-1.74)	-7.181	<0.001
Apo B	g/L	0.95 (0.76-1.17)	0.94 (0.73-1.14)	-0.613	0.540
Apo E*	mg/L	34.91 (27.94-40.84)	34.94 (29.30-42.85)	-1.091	0.275
Lp (a)	mg/L	141.85 (75.50-313.00)	101.30 (48.10-183.40)	-6.633	<0.001
DEIL	µmol/L	3.65 (2.80-5.00)	3.80 (3.00-5.00)	-0.262	0.793
IEIL	µmol/L	6.80 (5.20-9.40)	7.20 (5.40-9.70)	-0.232	0.817
PA*	mg/L	241.01 ± 59.95	249 ± 60.67	-2.496	0.013
GLB*	g/L	27.38 ± 4.69	27.85 ± 4.40	-1.481	0.139
ALB	g/L	41.17 ± 4.55	41.95 ± 4.19	-3.249	0.001
LDL-C	mmol/L	2.42 (1.81-2.98)	2.26 (1.79-2.84)	-1.316	0.188
HDL-C	mmol/L	1.07 (0.93-1.28)	1.16 (1.00-1.27)	-3.751	<0.001
TG	mmol/L	1.52 (1.07-2.01)	1.57 (1.17-2.20)	-0.220	0.826
TC	µmol/L	4.20 (3.42-4.90)	4.15 (3.54-4.98)	-0.215	0.829
TT	s	17.50 (16.80-18.30)	17.50 (16.80-18.40)	-0.431	0.667
PT	s	13.10 (12.70-13.50)	13.10 (12.70-13.50)	-1.085	0.278
INR		1.00 (0.95-1.04)	1.00 (0.96-1.04)	-1.426	0.154
FIB	g/L	3.48 (3.04-4.22)	3.38 (2.86-3.98)	-4.973	<0.001
Crea	µmol/L	70.15 (58.90-87.40)	63.00 (54.80-77.10)	-7.580	<0.001
Glu	mmol/L	8.35 (6.43-12.14)	7.86 (6.21-10.89)	-4.428	<0.001
HbA1c	%	7.84 (6.76-9.23)	7.08 (6.56-8.80)	-6.316	<0.001

The measurement data subject to normal distribution is represented by $\bar{x}$ *± s*, and the measurement data not subject to normal distribution is represented by *M (Q_25_, Q_75_)*; The enumeration data is expressed in n(%). * indicates missing value > 30%.

### Features in CHD were selected by six methods

**Figure 1:**
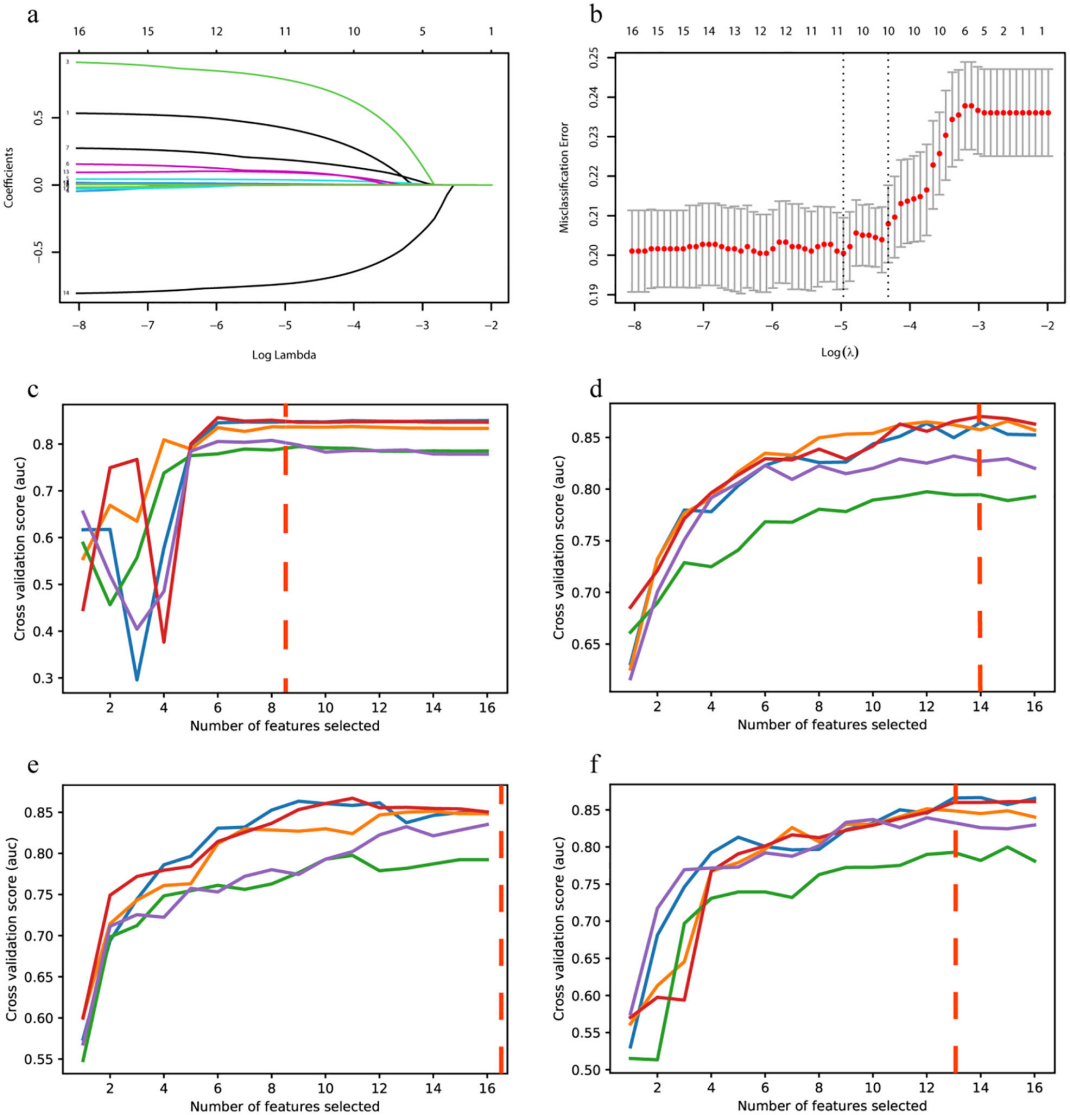
LASSO regression screening T2DM combined CHD features and RFE + 5-fold cross-validation screening features process. **(a)** 15 difference index model punishment process. **(b)** Optimal parameters in lasso regression model λ Change process. **(c)** RFE+SVM screening features process. **(d)** RFE +RF screening features process. **(e)** RFE+XgBoost screening features process. **(f)** RFE+LightGBM screening features process.

The features were further selected using the training set by six methods. Logistic regression identified eight features with *P*<0.05 (**Table 2**). The variation process of the penalty coefficient λ and index coefficient is shown in **Figure 1a**. The optimal λ value (**Figure 1b**, dashed line on the right) was determined to be within one variance range of the minimum mean square error (**Figure 1b**, dashed line on the left) through cross-validation. Ten features were selected by Lasso regression. Details of these features are shown in **Supplementary Table 4**.

**Table 2:** Logistic regression analysis results of difference index of T2DM complicated with CHD in training data.

Indicators	B	S.E	Wald χ ^2^	*P*	OR (95%CI)
Hypertension	0.540	0.144	14.140	<0.001	1.716 (1.295,2.274)
Smoke	0.927	0.188	24.219	<0.001	2.527 (1.747,3.655)
Age	0.044	0.007	37.417	<0.001	1.045 (1.031,1.060)
HbA1c	0.281	0.068	16.823	<0.001	1.324 (1.158,1.514)
AST	0.011	0.003	17.045	<0.001	1.011 (1.006,1.017)
Crea	0.015	0.004	15.759	<0.001	1.015 (1.008,1.023)
Lp (a)	0.004	<0.001	114.610	<0.001	1.004 (1.004,1.005)
Apo Ai	-0.815	0.330	6.113	0.013	0.443 (0.232,0.845)
Glu	-0.032	0.024	1.755	0.185	0.968 (0.924,1.015)
FIB	0.092	0.085	1.176	0.278	1.096 (0.928,1.294)
Urine Glu	0.160	0.169	0.894	0.344	1.173 (0.843,1.633)
ALB	-0.019	0.028	0.458	0.498	0.981 (0.929,1.037)
TP	0.009	0.015	0.329	0.566	1.009 (0.979,1.039)
Drink	-0.060	0.192	0.099	0.753	0.942 (0.647,1.371)
Diabetic nephropathy	0.028	0.329	0.007	0.932	1.028 (0.540,1.958)
HDL-C	0.004	0.316	<0.001	0.991	1.004 (0.540,1.865)
Constant	-5.749	1.298	19.630	<0.001	

**Figure 2:**
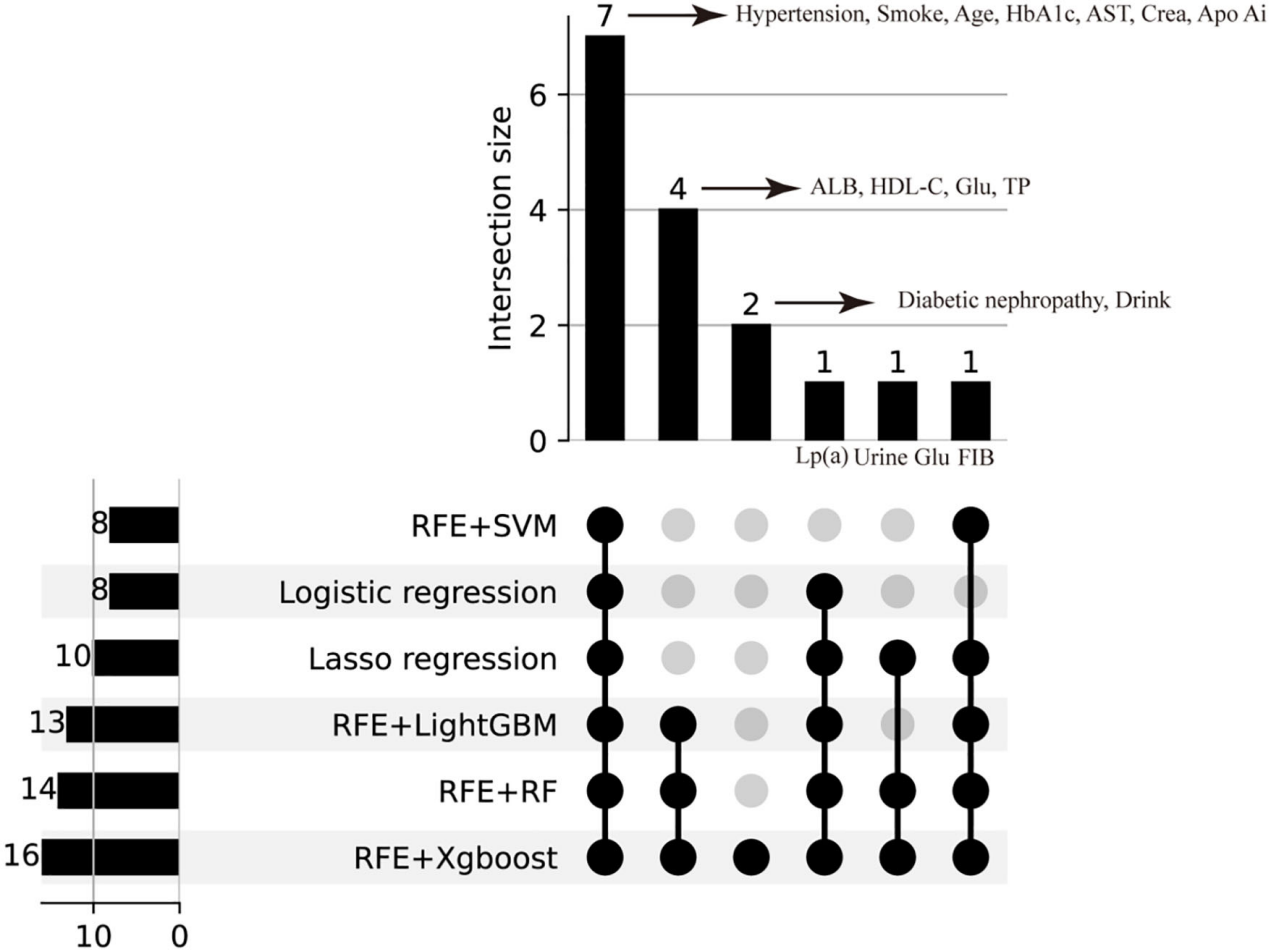
UpSet plot of overlapping features selected by multiple methods on the test set.

Four RFE underlying models, including SVM, RF, XgBoost, and LightGBM, were used in this study. To find the optimal features for each of these models, we employed 5-fold cross-validation to verify the maximum AUC in the training set. The screening process is shown in **Figures 1c-f**, where each line represents one validation in the 5-fold cross-validation, and the red dashed line represents the feature with the highest average AUC value. Eight features were selected by RFE+SVM (**Figure 1c**). Fourteen features were selected by RFE+RF (**Figure 1d**). Sixteen features were selected by RFE+XgBoost (**Figure 1e**). Thirteen features were selected by RFE+LightGBM (**Figure 1f**). Details of these features are shown in **Supplementary Table 4**. To better illustrate the intersection of selected features across different methods, we utilized an UpSet plot, which provides a clear and comprehensive visualization of feature overlaps (**Figure 2**).

### Establishment of classification models

Based on the six distinct feature selection methods, the corresponding clinical feature sets detailed in **Supplementary Table 4** were used as input variables for each classification model, with the output variable being the presence or absence of comorbid CHD, we established models based on Logistic Regression, SVM, RF, XgBoost, and LightGBM. We performed a 5-fold cross-validation on the training set and used the testing set to verify the performance of the classification models. Since the hyperparameter optimization in this study aimed to maximize the AUC, we compared the AUC values of the various models. The performance and ROC curve of each classification model, verified by 5-fold cross-validation based on six feature sets in the training set, are shown in **Supplementary Table 5**.

### Performance of classification models with different features selection

**Figure 3:**
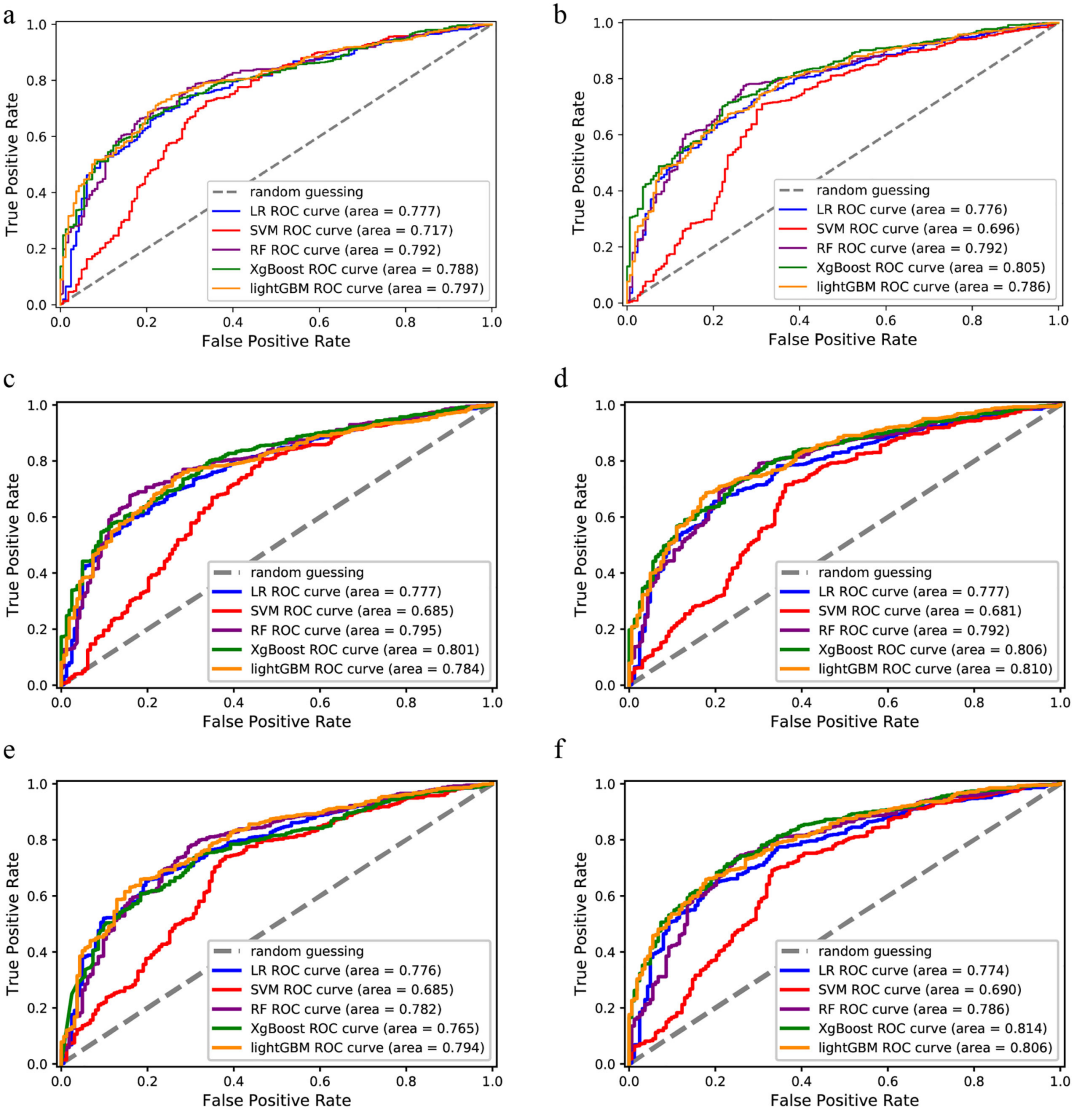
ROC curve of 5 classification models in validation set. **(a)** ROC curve of 5 classification models based on Logistic regression screening method in validation set. **(b)** ROC curve of 5 classification models based on Lasso regression screening method in validation set. **(a)** ROC curve of 5 classification models based on RFE + SVM variable screening method in validation set. **(d)** ROC curve of 5 classification models based on RFE + RF variable screening method in validation set. **(e)** ROC curve of 5 classification models based on RFE + XgBoost variable screening method in validation set. **(f)** ROC curve of 5 classification models based on RFE + LightGBM variable screening method in validation set. LR: Logistic regression.LR: Logistic regression.

After successfully establishing the models, To identify the optimal classification model, we further evaluated performance using the testing set to verify the performance of each classification model. The result was as follows (**Table 3**). The LightGBM model (AUC=0.797) achieved the best performance based on features selected by Logistic regression (**Figure 3a**). The XGBoost model (AUC=0.805) outperformed other models based on features selected by Lasso regression (**Figure 3b**). The XGBoost model, based on features selected by RFE+SVM, showed the best performance (AUC=0.801) (**Figure 3c**). The LightGBM model achieved the best performance based on features selected by RFE+RF (AUC=0.810) (**Figure 3d**) and RFE+XGBoost (AUC=0.794) (**Figure 3e**). The XGBoost model, based on features selected by RFE+LightGBM, obtained the highest AUC value (AUC = 0.814) compared to other models based on the six features (**Table 3** and **Figure 3f**). Thus, we demonstrated that XgBoost model based on RFE+LightGBM selected features is the optimal auxiliary diagnostic model.

**Table 3:** Performance evaluation table of 5 classification models in testing set.

Classification model	Accuracy *(95% CI)*	Precision *(95% CI)*	Recall *(95% CI)*	F1 Score *(95% CI)*	AUC *(95% CI)*
Feature selection method: Logistic regression					
Logistic regression	0.695 (0.662,0.727)	0.916 (0.890,0.941)	0.675 (0.638,0.712)	0.777 (0.750,0.804)	0.777 (0.737,0.814)
SVM	0.750 (0.719,0.781)	0.868 (0.840,0.895)	0.806 (0.774,0.837)	0.836 (0.813,0.858)	0.717 (0.667,0.764)
RF	0.768 (0.738,0.798)	0.890 (0.863,0.916)	0.806 (0.774,0.837)	0.846 (0.823,0.868)	0.792 (0.754,0.827)
XgBoost	0.736 (0.705,0.767)	0.905 (0.878,0.930)	0.744 (0.709,0.778)	0.816 (0.791,0.840)	0.788 (0.751,0.823)
LightGBM	0.751 (0.720,0.782)	0.905 (0.879,0.929)	0.765 (0.731,0.798)	0.829 (0.805,0.853)	0.797 (0.761,0.831)
Feature selection method: Lasso regression					
Logistic regression	0.693 (0.661,0.726)	0.908 (0.881,0.934)	0.680 (0.644,0.717)	0.778 (0.750,0.805)	0.776 (0.737,0.814)
SVM	0.762 (0.732,0.791)	0.853 (0.824,0.881)	0.843 (0.815,0.872)	0.848 (0.826,0.869)	0.696 (0.650,0.775)
RF	0.768 (0.738,0.798)	0.889 (0.862,0.914)	0.808 (0.776,0.838)	0.846 (0.824,0.868)	0.792 (0.753,0.828)
XgBoost	0.746 (0.715,0.777)	0.898 (0.872,0.924)	0.765 (0.731,0.798)	0.826 (0.802,0.850)	0.805 (0.769,0.838)
LightGBM	0.731 (0.701,0.763)	0.901 (0.874,0.926)	0.742 (0.707,0.777)	0.814 (0.788,0.838)	0.786 (0.747,0.822)
Feature selection method: RFE+SVM					
Logistic regression	0.787 (0.758,0.817)	0.837 (0.809,0.865)	0.907 (0.883,0.930)	0.871 (0.850,0.890)	0.777 (0.736,0.814)
SVM	0.762 (0.731,0.791)	0.851 (0.822,0.879)	0.847 (0.817,0.875)	0.849 (0.826,0.870)	0.685 (0.634,0.735)
RF	0.800 (0.772,0.829)	0.833 (0.805,0.860)	0.935 (0.914,0.954)	0.881 (0.862,0.899)	0.795 (0.757,0.832)
XgBoost	0.798 (0.769,0.826)	0.838 (0.810,0.866)	0.922 (0.900,0.942)	0.878 (0.858,0.896)	0.801 (0.765,0.835)
LightGBM	0.782 (0.753,0.811)	0.852 (0.824,0.880)	0.876 (0.849,0.902)	0.864 (0.843,0.884)	0.784 (0.747,0.820)
Feature selection method: RFE+RF					
Logistic regression	0.787 (0.758,0.817)	0.841 (0.813,0.869)	0.900 (0.876,0.924)	0.870 (0.850,0.889)	0.777 (0.737,0.815)
SVM	0.786 (0.756,0.814)	0.808 (0.779,0.837)	0.956 (0.939,0.971)	0.879 (0.857,0.894)	0.681 (0.631,0.730)
RF	0.808 (0.780,0.835)	0.818 (0.789,0.846)	0.974 (0.961,0.956)	0.889 (0.871,0.906)	0.792 (0.754,0.830)
XgBoost	0.796 (0.768,0.825)	0.847 (0.819,0.875)	0.905 (0.882,0.928)	0.875 (0.856,0.894)	0.806 (0.771,0.840)
LightGBM	0.805 (0.777,0.832)	0.861 (0.834,0.887)	0.900 (0.874,0.922)	0.879 (0.860,0.898)	0.810 (0.774,0.844)
Feature selection method: RFE+XgBoost					
Logistic regression	0.787 (0.758,0.817)	0.840 (0.812,0.868)	0.902 (0.878,0.925)	0.870 (0.850,0.890)	0.776 (0.736,0.814)
SVM	0.786 (0.758,0.814)	0.803 (0.774,0.832)	0.965 (0.950,0.979)	0.877 (0.858,0.895)	0.685 (0.636,0.732)
RF	0.800 (0.772,0.829)	0.815 (0.787,0.843)	0.966 (0.951,0.979)	0.884 (0.866,0.902)	0.782 (0.741,0.821)
XgBoost	0.790 (0.762,0.818)	0.796 (0.767,0.824)	0.987 (0.977,0.995)	0.881 (0.863,0.899)	0.765 (0.725,0.802)
LightGBM	0.796 (0.768,0.825)	0.859 (0.832,0.886)	0.887 (0.861,0.912)	0.873 (0.853,0.893)	0.794 (0.755,0.830)
Feature selection method: RFE+LightGBM					
Logistic regression	0.790 (0.760,0.818)	0.841 (0.812,0.868)	0.905 (0.882,0.928)	0.872 (0.852,0.891)	0.774 (0.734,0.812)
SVM	0.794 (0.764,0.822)	0.818 (0.789,0.850)	0.951 (0.933,0.967)	0.879 (0.860,0.897)	0.690 (0.639,0.738)
RF	0.796 (0.768,0.825)	0.811 (0.782,0.839)	0.967 (0.952,0.981)	0.882 (0.864,0.900)	0.786 (0.746,0.824)
XgBoost	0.799 (0.771,0.827)	0.841 (0.812,0.868)	0.920 (0.898,0.941)	0.879 (0.859,0.897)	0.814 (0.779,0.847)
LightGBM	0.803 (0.774,0.830)	0.848 (0.820,0.875)	0.914 (0.891,0.935)	0.880 (0.860,0.898)	0.806 (0.771,0.839)

### Visualization of feature importance

**Figure 4:**
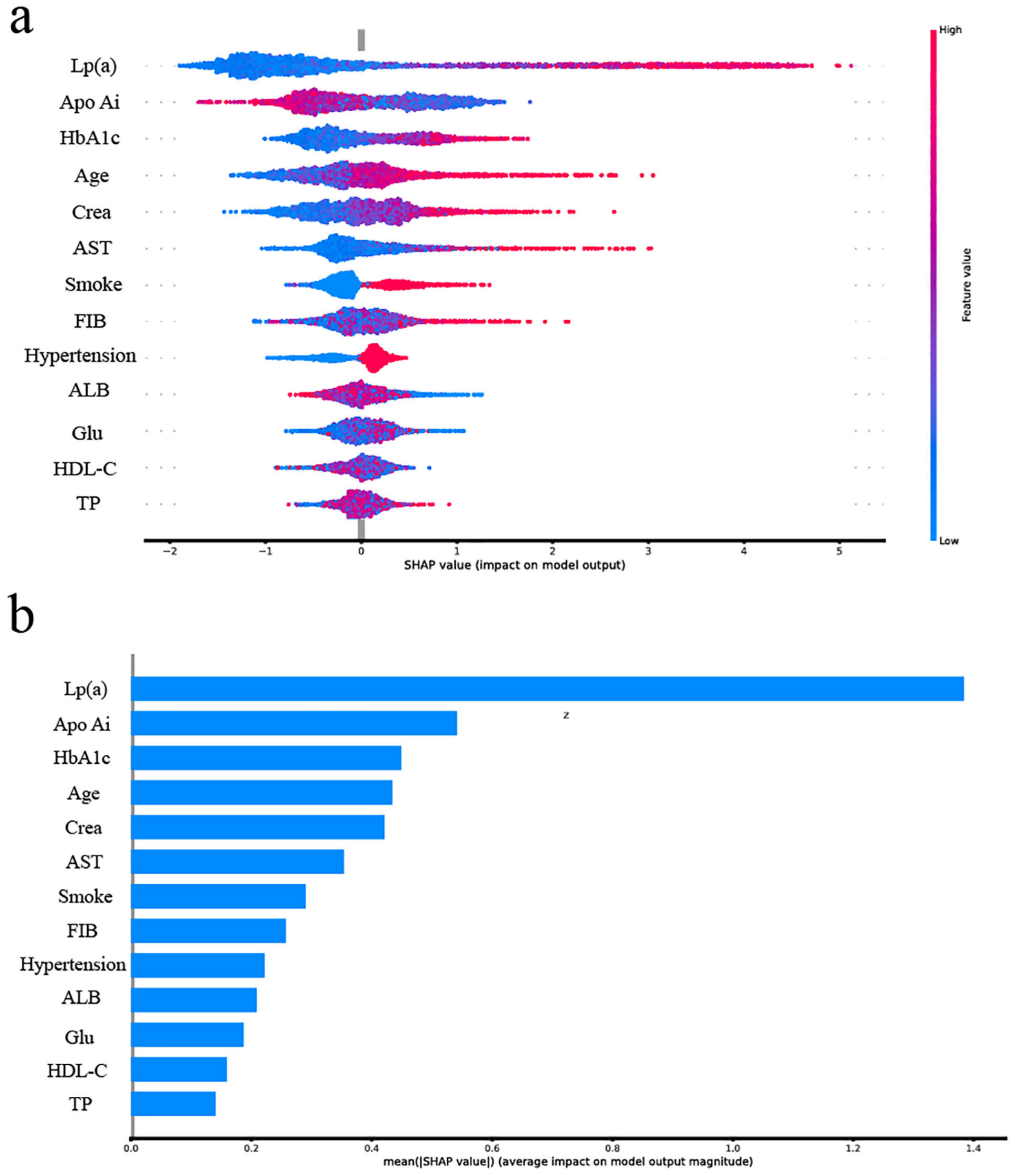
The importance of feature in optimized. **(a)** SHAP value of each feature in the optimal model. SHAP summary plot showing the contribution of each feature to the prediction of CHD risk. Each point represents a sample, with color indicating the actual feature value (red = high, blue = low). The x-axis represents the SHAP value, which reflects the direction and magnitude of the feature’s impact on CHD prediction. Positive SHAP values (right side) indicate increased risk, while negative values (left side) indicate decreased risk. For example, if red points are predominantly located on the right, it suggests that higher values of the feature are associated with increased CHD risk. **(b)** Average SHAP value of each feature in the optimal model.

To intuitively explain the selected features, we used SHAP to calculate their importance in determining whether T2DM patients also have CHD. As shown in **Figure 4a**, higher values of features such as HbA1c, Crea, AST, Lp(a), Apo Ai, hypertension, smoking status, age, fibrinogen (FIB), HDL-C, albumin (ALB), glucose (Glu) and total protein (TP). The thirteen features in the optimal auxiliary diagnostic model were ranked by their average SHAP values. The feature ranking on the y-axis indicates their importance in the auxiliary diagnostic model (**Figure 4b**).

## Discussion

Our study demonstrates that routinely collected clinical variables can be effectively leveraged to distinguish T2DM patients with CHD from those without, using machine learning-based models. Among the models tested, the XGBoost classifier using features selected via recursive feature elimination combined with LightGBM (RFE+LightGBM) achieved the highest performance (AUC = 0.814), indicating strong discriminatory ability. The reliance on readily accessible clinical indicators, such as routine biochemical and hematological parameters, enhances the model’s feasibility for real-world application, particularly in endocrinology and primary care settings where cardiovascular risk in T2DM patients is frequently underrecognized. This approach offers potential value for the early identification of CHD and the timely initiation of secondary prevention strategies to reduce the risk of major cardiovascular events.

In contrast to many prior studies that applied machine learning to CHD prediction but suffered from key limitations—such as heterogeneous or unspecified populations, unclear outcome definitions in control groups, and lack of objective CHD validation (20–22). Our study addressed these issues systematically. Specifically, we enrolled a well-defined cohort of T2DM patients who all underwent CA, enabling objective classification into CHD and non-CHD groups based on a standardized criterion of ≥50% stenosis in one or more major coronary arteries. This design improves both diagnostic precision and population homogeneity. Compared with recent work by Sang et al. (23) who developed a CHD prediction model in T2DM patients based on EHR data and achieved an AUROC of 0.722 using random forest algorithms, our study offers several improvements. These include the use of angiographically confirmed CHD diagnoses, a higher model performance (AUC=0.814), and a broader range of feature selection strategies. Furthermore, our model’s interpretability and clinical integration potential are enhanced by its foundation in real-world, routinely collected data.

Additionally, we applied six independent feature selection techniques alongside five machine learning classifiers to rigorously explore the optimal predictive strategy. This multi-method approach reduced selection bias and improved model generalizability.

The training set is primarily used for features selection and classification model training, so when evaluating model performance, the focus is mainly on using the testing set for assessment. In this study, model optimization is based on maximizing the AUC value, so the performance of the thirty established models in the testing set is primarily evaluated through the AUC value. **Supplementary Figure S2** shows a comparison of the testing set AUC values for 6 different variable screening methods applied to 5 classification models. As seen in **Supplementary Figure S2a**, the model performance of Logistic regression does not vary significantly across these 6 variable screening methods. Whether using RFE+XgBoost with up to 16 features or Logistic regression with 8 features, the AUC values on the training set differ only by a fraction of a percentage. Therefore, when using Logistic regression to build classification models for disease prediction, it may be preferable to use a variable screening method with fewer features. **Supplementary Figure S2b** shows the performance of classification models established using the SVM algorithm based on 6 different variable screening methods. This study found that the Logistic regression is optimal and significantly outperforms the other methods. However, as shown in **Supplementary Figure S3a**, the SVM algorithm exhibits the poorest classification performance among the 5 models in this study. **Supplementary Figure S2c** shows the AUC values of the RF algorithm under 6 variable screening methods. The study found that the RF algorithm performs better with the Logistic regression, Lasso regression, and RFE+SVM variable screening methods compared to the other three methods. This features that the RF model is not well-suited for using a larger number of indicators and tends to achieve better results with fewer features. **Supplementary Figures S1d, e** show the AUC values of the XgBoost and LightGBM algorithms under 6 different variable screening methods. Except for the RFE+XgBoost method, which does not remove any variables, using more indicators with the other 5 variable screening methods generally leads to better classification performance with these two machine learning models. Specifically, the classification model established using the XgBoost algorithm based on the RFE+LightGBM variable screening methods performed the best in this study (AUC=0.814).

As shown in **Supplementary Figure S3a**, the XgBoost model (average AUC=0.797) and the LightGBM model (average AUC=0.796) outperform the other three classification models in this study. These two models are similar, which aligns with their underlying principles. The LightGBM algorithm mainly surpasses XgBoost in training speed and memory usage, while their performance in terms of model accuracy is comparable. As seen in **Supplementary Figure S3b**, the Logistic regression and RFE+LightGBM methods are the most optimal variable screening methods. However, a closer comparison reveals that the superior performance of the Logistic regression variable screening methods is mainly due to the large difference between the best and second-best models when using the SVM model. **Supplementary Figure S3c** shows the average AUC values of the 6 variable screening methods after excluding the SVM model. Apart from the RFE+XgBoost method, which includes all variables, the average AUC values of the other selection methods are positively correlated with the number of variables included. The suboptimal performance of the RFE+XgBoost method may be due to its overly complex algorithm, which consumes a large amount of memory. This could prevent the method from effectively selecting useful variables, resulting in lower AUC values on the subsequent training set.

Our study identified thirteen features: HbA1c, Crea, AST, Lp(a), Apo Ai, hypertension, smoking status, age, FIB, HDL-C, ALB, Glu and TP. As shown in the Venn diagram (**Supplementary Figure S3d**) based on RFE, Lasso regression, and logistic regression, seven features including, HbA1c, Crea, AST, Lp(a), hypertension, smoking status and age, were consistently selected by all six methods and have proven significant in diagnostic models. This result further confirms the importance of these features in the auxiliary diagnosis model for CHD in T2DM patients.

HbA1c is one of the diagnostic criteria for T2DM, recent studies have shown its association with cardiovascular risk (24), which is consistent with our findings. Crea is commonly used in clinical practice to assess kidney function. Literature indicates that reduced kidney function increases CHD risk. Agus et al. (25) conducted a case-control study in non-DM patients and found that adding Crea to traditional risk factors improved CHD risk prediction, which is consistent with our findings.AST reflects the severity of myocardial cell damage. Several studies (26–28) suggest that AST should be included in various CHD risk prediction models. In this study, AST was identified by all six screening methods, indicating its potential as a biomarker for differentiating CHD in T2DM patients. It is still unclear whether Lp(a) is a protective factor or a risk factor for T2DM (29), but it is certain that abnormal Lp(a) levels in T2DM are noteworthy.

Notably, Apo Ai, the second most important feature in the optimal model, was not identified in **Supplementary Figure S3d**. However, Apo Ai, the main protein component of HDL-C, has anti-atherosclerotic effects (30). **Figure 3a** shows that lower Apo Ai values are associated with higher CHD probability, consistent with **Supplementary Table 4** and reviews of Apo Ai (31). Hypertension, smoking status and age have been reported as CHD risk factors in T2DM patients (32). The other features in the optimized model, FIB and HDL-C, are consistent with previous studies (30, 33). A meta-analysis found a non-linear relationship between Glu and cardiovascular disease in non-T2DM patients (34). There are few studies comparing ALB and TP levels in T2DM patients with and without CHD.

Despite the strengths of our study, several limitations should be acknowledged. First, this was a retrospective study based on electronic medical records from a single medical data platform, which may limit the generalizability of our findings to broader or more diverse populations. Second, although CA provides an objective standard for CHD diagnosis, the dataset lacked longitudinal follow-up information, preventing us from evaluating the prognostic value of the model over time. Third, we did not incorporate treatment-related variables, such as medication history or lifestyle interventions, which may have influenced both feature distribution and CHD risk. Lastly, external validation using independent datasets from other institutions is still warranted to confirm the robustness and clinical applicability of our predictive model.

Future research should focus on addressing these limitations. Expanding the model to include multicenter cohorts with more diverse patient populations will enhance its external validity. Additionally, incorporating longitudinal follow-up data will allow for evaluation of the model’s prognostic value in predicting long-term cardiovascular outcomes. Integrating treatment variables, such as medication use and lifestyle factors, may further improve the clinical relevance and accuracy of the model. Finally, prospective studies and real-world implementation are necessary to assess how the model performs in routine clinical practice and whether it can serve as a reliable decision support tool for early CHD risk stratification in T2DM patients.

## Conclusion

We have developed a XgBoost classification model based on T2DM patient electronic medical record data to determine whether patients are developing CHD. Moreover, based on the final model, we found that Lp (a), Apo Ai, HbA1c, Age, Crea, AST and other features are important in determining whether T2DM patients have CHD. Our approach addresses key limitations in previous CHD risk prediction studies by utilizing coronary angiography-verified diagnoses and a well-defined diabetic population. The model’s reliance on accessible clinical indicators enhances its feasibility for early risk stratification, particularly in primary care and endocrinology settings. These findings contribute to the growing body of research supporting the use of data-driven tools in chronic disease management and provide a foundation for future work in precision cardiovascular prevention.

## Data Availability

The original contributions presented in the study are included in the article/**Supplementary Material**. Further inquiries can be directed to the corresponding author.
