# Supplementary material for: Machine learning-based coronary heart disease diagnosis model for type 2 diabetes patients
Source: Front Endocrinol (Lausanne). 2025 May 22;16:1550793. doi: 10.3389/fendo.2025.1550793 (PMC12137098; doi:10.3389/fendo.2025.1550793)
Supplement: Supplementary file 1 [file DataSheet1.docx]

**Supplementary Tab. 1 The description of the study population**

| Description of Variables | Enrolled patients(n=2517) | Missing Percentage(%) |
| --- | --- | --- |
| Demographic information |  |  |
| Age(year) | 66.00(60.00-73.00) | 0.00% |
| Male,n(%) | 1417(56.30%) | 0.00% |
| BMI(kg/m^2^) | 24.97(23.12-27.35) | 75.80% |
| Smoke,n(%) | Yes 882(36.86%) | 4.93% |
|  | No 1511(63.14%) |  |
| Drink,n(%) | Yes 640(26.86%) | 5.32% |
|  | No 1743(73.14%) |  |
| Systolic pressure (mmHg) | 138.00(125.00-151.00) | 4.69% |
| Diastolic pressure (mmHg) | 80.00(72.00-90.00) | 4.65% |
| Heart rate (beats/min) | 78.00(70.00-87.00) | 4.65% |
| Family history of diabetes,n(%) | 246(9.77%) | 0.00% |
| Family history of CHD,n(%) | 106(4.21%) | 0.00% |
| Indications |  |  |
| Coronary heart disease,n(%) | 1942(77.16%) | 0.00% |
| Hypertension,n(%) | 1793(71.24%) | 0.00% |
| Diabetic nephropathy,n(%) | 164(6.52%) | 0.00% |
| Cerebral infarction,n(%) | 352(13.98%) | 0.00% |
| Carotid atherosclerosis,n(%) | 428(17.00%) | 0.00% |
| Atrial fibrillation,n(%) | 112(4.45%) | 0.00% |
| Heart block,n(%) | 75(2.98%) | 0.00% |
| Hyperlipidemia,n(%) | 696(27.65%) | 0.00% |
| Laboratory results |  |  |
| Urine Glu,n(%) | Positive 844(39.18%) | 10.37% |
|  | Negative 1372(60.82%) |  |
| Urine protein,n(%) | Positive 381(21.78%) | 30.51% |
|  | Negative 1368(78.22%) |  |
| Urine WBC#(/µL) | 4.65(1.90-16.20) | 35.88% |
| Urine RBC#(/µL) | 5.90(2.80-12.10) | 34.13% |
| Urine Crea(mmol/L) | 8.80(4.40-17.60) | 53.04% |
| ALT(U/L) | 23.00(15.40-35.00) | 6.75% |
| AST(U/L) | 22.00(17.00-31.00) | 6.28% |
| GGT(U/L) | 29.00(20.00-49.00) | 16.61% |
| TBIL(µmol/L) | 10.70(8.20-14.43) | 6.79% |
| TP(g/L) | 69.66±6.99 | 19.94% |
| Apo Ai(g/L) | 1.39(1.19-1.62) | 25.31% |
| Apo B(g/L) | 0.95(0.75-1.17) | 25.31% |
| Apo E(mg/L) | 35.20(28.93-42.40) | 41.76% |
| Lp(a)(mg/L) | 135.05(66.18-281.78) | 26.23% |
| DEIL(µmol/L) | 4.00(2.80-5.30) | 8.10% |
| IEIL(µmol/L) | 6.80(5.00-9.31) | 8.22% |
| PA(mg/L) | 243.05±60.22 | 31.98% |
| GLB(g/L) | 27.48±4.63 | 34.06% |
| ALB(g/L) | 41.34±4.48 | 19.94% |
| LDL-C(mmol/L) | 2.51(1.90-3.13) | 10.53% |
| HDL-C(mmol/L) | 1.07(0.90-1.28) | 9.14% |
| TG(mmol/L) | 1.59(1.13-2.25) | 9.14% |
| TC(µmol/L) | 4.39(3.64-5.19) | 9.14% |
| TT(s) | 17.10(15.90-18.10) | 6.08% |
| PT(s) | 12.70(11.60-13.30) | 4.77% |
| INR | 0.98(0.93-1.03) | 4.77% |
| FIB(g/L) | 3.29(2.78-3.89) | 5.84% |
| Crea(µmol/L) | 70.10(58.00-85.60) | 17.56% |
| Glu(mmol/L) | 8.06(6.41-11.30) | 29.60% |
| HbA1c(%) | 7.30(6.50-8.48) | 18.75% |

The measurement data subject to normal distribution is represented by *x±s*, and the measurement data not subject to normal distribution is represented by *M (P_25_, p_75_)*; The enumeration data is expressed in n(%).

Supplementary Tab. 2 Variables and their assignments

| Characteristic | Assignment |
| --- | --- |
| Gender | Male=1; Female=0 |
| Smoke | Yes=1; No=0 |
| Drink | Yes=1; No=0 |
| Family history of diabetes | Yes=1; No=0 |
| Family history of CHD | Yes=1; No=0 |
| Coronary heart disease | Combined=1; No=0 |
| Hypertension | Combined=1; No=0 |
| Diabetic nephropathy | Combined=1; No=0 |
| Cerebral infarction | Combined=1; No=0 |
| Carotid atherosclerosis | Combined=1; No=0 |
| Atrial fibrillation | Combined=1; No=0 |
| Heart block | Combined=1; No=0 |
| Hyperlipidemia | Combined=1; No=0 |
| Urine glucose | Negative=0；Positive=1 |
| Urine protein | Negative=0；Positive=1 |

**Supplementary Tab. 3 comprehensive statistical comparison between the pre- and post-imputation datasets**

| Variable | Measurement units | Before(n=2517) | After(n=2517) | Test | p-value |
| --- | --- | --- | --- | --- | --- |
| Hypertension | n% | 1793(71.24%) | 1793(71.24%) | Chi-square | 1 |
| Drink | n% | 640(26.86%) | 672(26.70%) | Chi-square | 0.094 |
| Smoke | n% | 882(36.86%) | 905(35.96%) | Chi-square | 0.571 |
| Urine Glu | n% | 844(39.18%) | 971(38.58%) | Chi-square | 0.090 |
| Diabetic nephropathy | n% | 164(6.52%) | 164(6.52%) | Chi-square | 1 |
| Apo Ai | g/L | 1.39(1.19-1.62) | 1.37(1.20-1.56) | Mann-Whitney U | 0.300 |
| Glu | mmol/L | 8.06(6.41-11.30) | 8.31(6.81-11.20) | Mann-Whitney U | 0.376 |
| AST | U/L | 22.00(17.00-31.00) | 22.63(17.10-31.80) | Mann-Whitney U | 0.139 |
| ALB | g/L | 41.42(38.50-44.23) | 41.70(38.8-44.39) | Mann-Whitney U | 0.147 |
| FIB | g/L | 3.29(2.78-3.89) | 3.27(2.79-3.85) | Mann-Whitney U | 0.900 |
| HDL-C | mmol/L | 1.07(0.90-1.28) | 1.07(0.91-1.26) | Mann-Whitney U | 0.788 |
| Crea | µmol/L | 70.10(58.00-85.60) | 71.40(60.40-84.70) | Mann-Whitney U | 0.070 |
| Age | year | 66.00(60.00-73.00) | 66.00(60.00-73.00) | Mann-Whitney U | 1 |
| Lp(a) | mg/L | 135.05(66.18-281.78) | 137.40(70.4-294.8) | Mann-Whitney U | 0.051 |
| TP | g/L | 69.73(65.62-73.61) | 70.20(65.80-73.71) | Mann-Whitney U | 0.142 |
| HbA1c | % | 7.30(6.50-8.48) | 7.30(6.60-8.43) | Mann-Whitney U | 0.308 |

Supplementary Tab. 4 The features selected by RFE + 5-fold cross-validation

| Underlying models | Selected features |
| --- | --- |
| Logistic regression | Hypertension, Smoke, Age, HbA1c, AST, Crea, Lp(a), Apo Ai |
| Lasso regression | Hypertension, Smoke, Age, Urine Glu, Glycated hemoglobin A1c (HbA1c), AST, creatinine (Crea), FIB, Apo Ai, and Lp(a) |
| RFE+SVM | Hypertension, Smoke, Age, HbA1c, AST, Crea, Apo Ai, FIB |
| RFE+RF | Hypertension, Smoke, Age, Urine Glu, HbA1c, AST, ALB, HDL-C, Crea, Glu, FIB, Apo Ai, Lp(a), TP |
| RFE+Xgboost | Hypertension, Diabetic nephropathy, Smoke, Drink, Age, Urine Glu, HbA1c, AST, ALB, HDL-C, Crea, Glu, FIB, Apo Ai, Lp(a), TP |
| RFE+lighGBM | Hypertension, Smoke, Age, HbA1c, AST, ALB, HDL-C, Crea, Glu, FIB, Apo Ai, Lp(a), TP |

Supplementary Tab. 5 Performance of 5 classification models verified by 5-fold cross-validation in training set

| Classification model | Accuracy | Precision | Recall | F1 Score | AUC |
| --- | --- | --- | --- | --- | --- |
| **Feature selection method: Logistic regression** | | | | | |
| Logistic regression | 0.783±0.004 | 0.787±0.004 | 0.982±0.003 | 0.874±0.002 | 0.828±0.022 |
| SVM | 0.765±0.011 | 0.836±0.004 | 0.861±0.019 | 0.848±0.009 | 0.736±0.045 |
| RF | 0.806±0.017 | 0.825±0.015 | 0.948±0.007 | 0.880±0.009 | 0.837±0.030 |
| XgBoost | 0.805±0.024 | 0.828±0.015 | 0.939±0.015 | 0.880±0.014 | 0.837±0.026 |
| lightGBM | 0.785±0.012 | 0.838±0.012 | 0.890±0.019 | 0.863±0.008 | 0.818±0.023 |
| **Feature selection method: Lasso regression** | | | | | |
| Logistic regression | 0.786±0.003 | 0.790±0.003 | 0.980±0.005 | 0.875±0.002 | 0.826±0.026 |
| SVM | 0.779±0.012 | 0.825±0.012 | 0.904±0.003 | 0.862±0.006 | 0.740±0.032 |
| RF | 0.805±0.012 | 0.817±0.006 | 0.960±0.009 | 0.883±0.007 | 0.845±0.028 |
| XgBoost | 0.809±0.018 | 0.843±0.012 | 0.922±0.014 | 0.880±0.011 | 0.842±0.030 |
| lightGBM | 0.787±0.012 | 0.848±0.015 | 0.880±0.014 | 0.863±0.007 | 0.801±0.028 |
| **Feature selection method: RFE+SVM** | | | | | |
| Logistic regression | 0.801±0.014 | 0.832±0.010 | 0.926±0.011 | 0.877±0.009 | 0.835±0.021 |
| SVM | 0.768±0.013 | 0.838±0.005 | 0.863±0.020 | 0.850±0.010 | 0.736±0.045 |
| RF | 0.806±0.019 | 0.824±0.016 | 0.948±0.009 | 0.882±0.011 | 0.835±0.030 |
| XgBoost | 0.801±0.010 | 0.830±0.012 | 0.930±0.007 | 0.877±0.005 | 0.837±0.028 |
| lightGBM | 0.790±0.015 | 0.848±0.016 | 0.883±0.017 | 0.865±0.009 | 0.804±0.023 |
| **Feature selection method: RFE+RF** | | | | | |
| Logistic regression | 0.800±0.016 | 0.833±0.011 | 0.924±0.013 | 0.876±0.010 | 0.828±0.028 |
| SVM | 0.779±0.007 | 0.790±0.003 | 0.969±0.010 | 0.870±0.005 | 0.720±0.029 |
| RF | 0.802±0.020 | 0.808±0.013 | 0.973±0.010 | 0.883±0.011 | 0.840±0.027 |
| XgBoost | 0.805±0.018 | 0.840±0.011 | 0.920±0.014 | 0.878±0.012 | 0.841±0.028 |
| lightGBM | 0.801±0.014 | 0.846±0.017 | 0.905±0.004 | 0.874±0.008 | 0.837±0.027 |
| **Feature selection method: RFE+XgBoost** | | | | | |
| Logistic regression | 0.800±0.014 | 0.830±0.008 | 0.928±0.014 | 0.876±0.009 | 0.827±0.029 |
| SVM | 0.776±0.007 | 0.784±0.006 | 0.976±0.008 | 0.869±0.004 | 0.710±0.038 |
| RF | 0.793±0.010 | 0.800±0.008 | 0.974±0.005 | 0.877±0.006 | 0.839±0.026 |
| XgBoost | 0.775±0.004 | 0.776±0.002 | 0.992±0.003 | 0.871±0.002 | 0.800±0.026 |
| lightGBM | 0.784±0.013 | 0.842±0.016 | 0.883±0.004 | 0.862±0.007 | 0.809±0.020 |
| **Feature selection method: RFE+lightGBM** | | | | | |
| Logistic regression | 0.800±0.014 | 0.831±0.009 | 0.926±0.010 | 0.876±0.008 | 0.829±0.026 |
| SVM | 0.785±0.010 | 0.799±0.007 | 0.960±0.015 | 0.872±0.006 | 0.736±0.022 |
| RF | 0.803±0.015 | 0.807±0.009 | 0.974±0.009 | 0.883±0.009 | 0.839±0.029 |
| XgBoost | 0.803±0.019 | 0.836±0.016 | 0.923±0.015 | 0.877±0.011 | 0.846±0.030 |
| lightGBM | 0.800±0.017 | 0.842±0.016 | 0.909±0.008 | 0.874±0.010 | 0.838±0.028 |


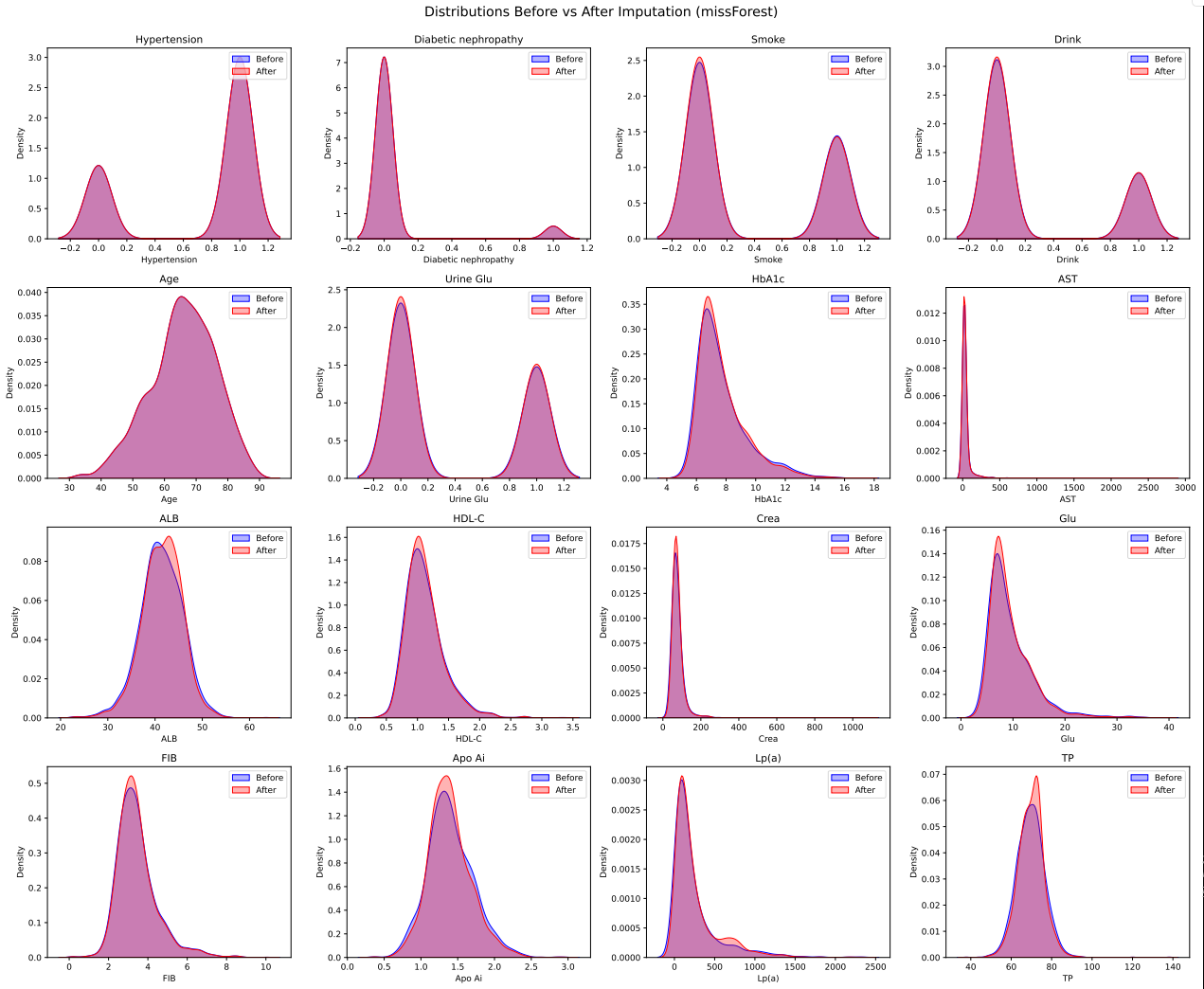


Fig.S1 Kernel density plots comparing the distributions of variables before and after imputation.

**
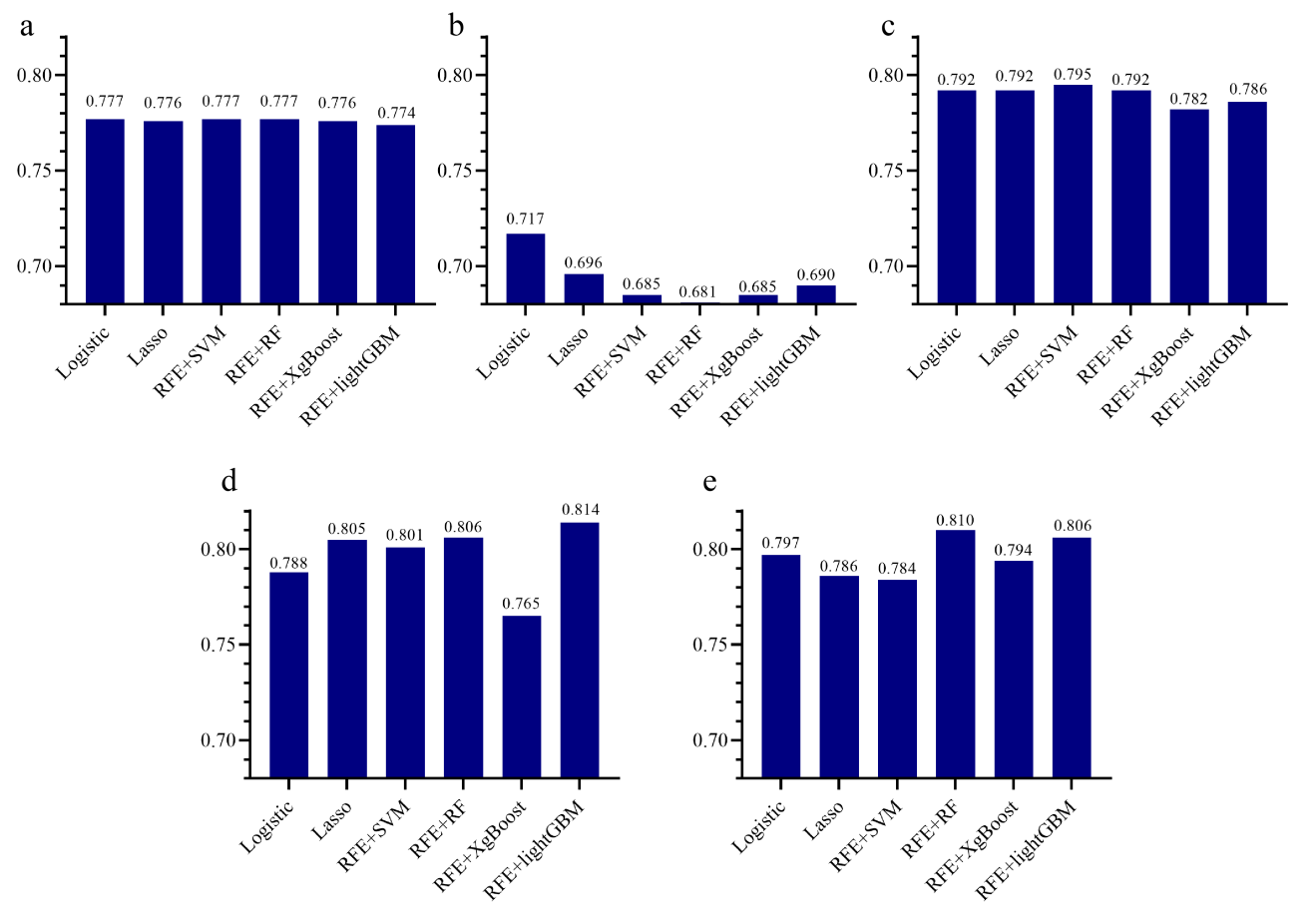
**

Fig.S2 AUC comparison of training sets of 6 different screening variable methods on 5 classification models. (a) 6 different methods of screening variables were used to establish the logistic regression model and compare the AUC values in the testing set. (b) 6 different methods of screening variables were used to establish the SVM model and compare the AUC values in the testing set. (c) 6 different methods of screening variables were used to establish the RF model and compare the AUC values in the testing set. (d) 6 different methods of screening variables were used to establish the XgBoost model and compare the AUC values in the testing set. (e) 6 different methods of screening variables were used to establish the lightGBM model and compare the AUC values in the testing set.


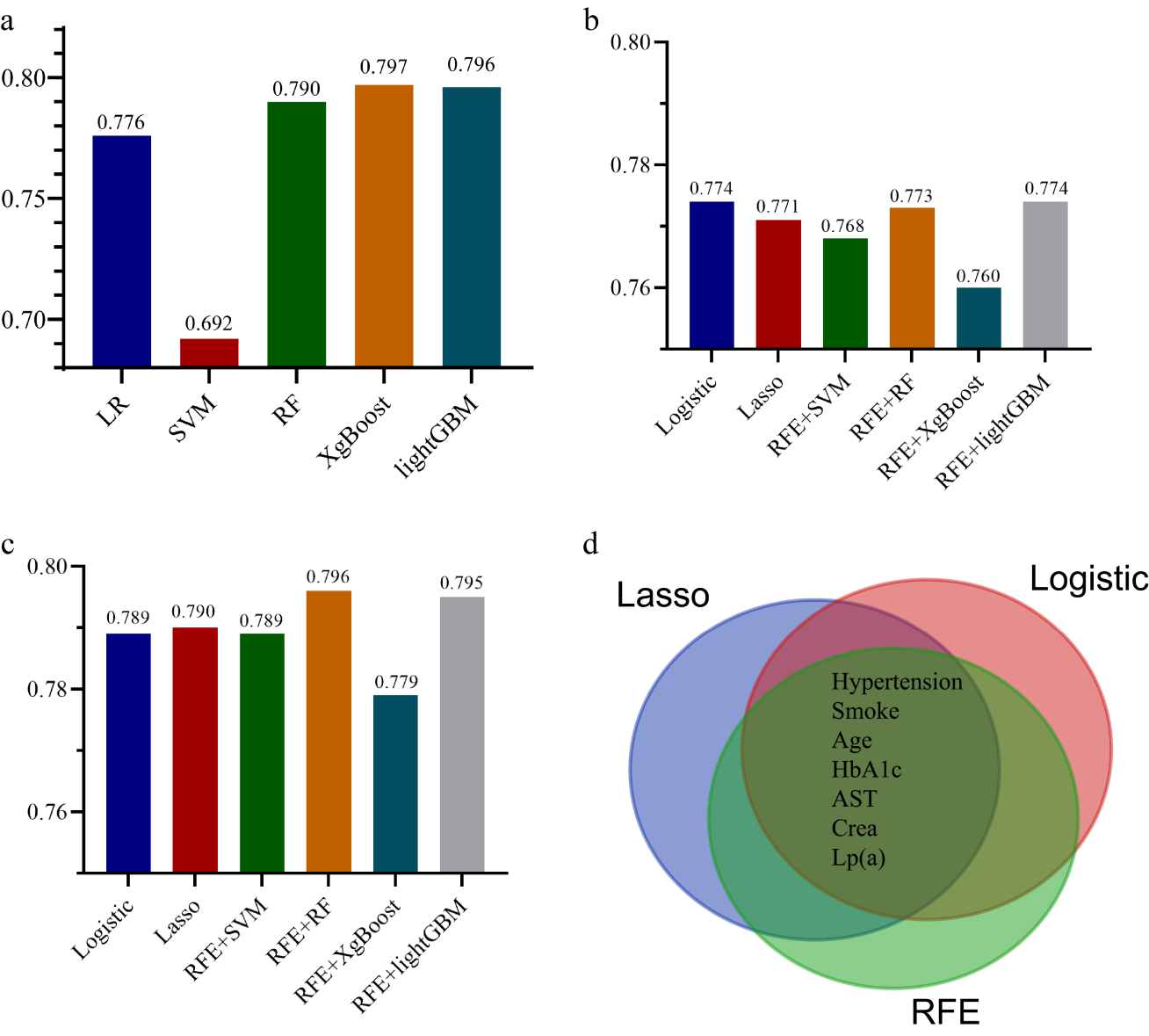


Fig.S3 average AUC value in testing set and Venn diagram of 6 variable screening methods. (a) average AUC value in testing set. (b) The average AUC of 5 Classification Models. (c) The average AUC of the other 4 classification models without SVM model. (d) Venn diagram of 6 variable screening methods.
